# Protein, Amino Acid, Oil, Fatty Acid, Sugar, Anthocyanin, Isoflavone, Lutein, and Antioxidant Variations in Colored Seed-Coated Soybeans

**DOI:** 10.3390/plants10091765

**Published:** 2021-08-25

**Authors:** Sanjeev Kumar Dhungana, Jeong-Hyun Seo, Beom-Kyu Kang, Ji-Hee Park, Jun-Hoi Kim, Jung-Sook Sung, In-Youl Baek, Sang-Ouk Shin, Chan-Sik Jung

**Affiliations:** Upland Crop Breeding Research Division, Department of Southern Area Crop Science, National Institute of Crop Science, Rural Development Administration, Miryang 50424, Korea; sanjeev@korea.kr (S.K.D.); hellobk01@korea.kr (B.-K.K.); heeya91@korea.kr (J.-H.P.); itomi123@korea.kr (J.-H.K.); sjs31@korea.kr (J.-S.S.); baekiy@korea.kr (I.-Y.B.); shinso32@korea.kr (S.-O.S.); jung100@korea.kr (C.-S.J.)

**Keywords:** antioxidant potential, colored seed coat, flavonoid, nutrient content, polyphenol, soybean

## Abstract

Different physiological and genetic studies show that the variations in the accumulation of pigment-stimulating metabolites result in color differences in soybean seed coats. The objective of this study was to analyze the nutrient contents and antioxidant potential in black, brown, and green seed-coated soybeans. Significant variations in protein (38.9–43.3%), oil (13.9–20.4%), total sugar (63.5–97.0 mg/g seed), total anthocyanin (3826.0–21,856.0 μg/g seed coat), total isoflavone (709.5–3394.3 μg/g seed), lutein (1.9–14.8 μg/g), total polyphenol (123.0–385.8 mg gallic acid/100 g seed), total flavonoid (22.1–208.5 mg catechin/100 g seed), 2,2′-azino-bis(3-ethylbenzthiazoline-6-sulphonic acid (ABTS; 275.0–818.8 mg Trolox/100 g seed), and 2,2-diphenyl-1-picrylhydrazyl (DPPH; 96.3–579.7 mg Trolox/100 g seed) were found among the soybean genotypes. Ilpumgeomjeong2 contained the lowest protein but the highest oil and total sugar. The lowest oil-containing Wonheug had the highest protein content. Socheong2 was rich in all four variables of antioxidants. Anthocyanins were detected only in black soybeans but not in brown and green soybeans. The variation in isoflavone content was up to 5-fold among the soybean genotypes. This study could be a valuable resource for the selection and improvement of soybean because an understanding of the nutrient content and antioxidant potentials is useful to develop effective strategies for improving the economic traits; for example, the major emphasis of soybean breeding for fatty acids is to enhance the oleic and linoleic acid contents and to decrease linolenic acid content.

## 1. Introduction

The demand for soybean (*Glycine max* (L.) Merr.), one of the top five largely produced crops globally, is increasing in the food, pharmaceutical, cosmetic, and biofuel industries [1,2] owing to their abundant protein, lipids, and several phytochemicals, including availability of isoflavones, anthocyanins, phenolics, and saponins [3,4,5,6]. Soybeans are available in a range of colors from yellow, green, brown, and black, to mottled [7]. Physiological and genetic studies indicate that the color variation is due to the difference in the accumulation of pigment-stimulating metabolites [8,9,10,11]. 

Black soybeans have been widely used in Asian countries, particularly as a folk medicine in China, Japan, and Korea for hundreds of years [12,13]. Studies have shown that anthocyanins are highly concentrated in black soybeans than in other soybeans [12,14,15]. The high level of anthocyanin content imparts to the better pharmacological capacities of black soybeans than other colored soybeans [16,17]. 

Both black and brown soybeans contain abundant flavan-3-ols, including catechin and epicatechin, which are the major antioxidants in brown-seeded soybean [18]. Catechins have drawn attention owing to their potential roles in several activities, including antibacterial [19], anti-inflammatory [20], and antioxidative activities [21,22], and could also possess the anti-carcinogenic effect [23]. Brown soybeans are rich in β-carotene compared to black, green, yellow, and mottled soybeans [24]. β-carotene is reported to have antioxidative and anticancer effects [25,26]. 

Green seed-coated soybeans contain higher total tannins than black and brown soybeans [27]. Sharma et al. [28] have reviewed that despite a few negative health consequences like anti-nutritional effect, reduced digestibility, mutagenic, and carcinogenic effects, tannin has recently been reported to have numerous health benefits such as anti-oxidant, anti-cancerous, anti-allergic, anti-inflammatory, anti-helminthic, and anti-microbial activities. 

Isoflavones have been measured in soybeans of all colors [14]. Soy isoflavones possess different pharmacological properties such as antioxidant, anti-inflammatory, anti-obesity, anti-diabetic, and antiviral activities [29,30,31]. Several studies have been carried out to examine different nutrient components and antioxidant potentials of soybeans of different seed coat colors. For instance, anthocyanin, isoflavone, and antioxidant activity of Korean black soybean landraces [32]; isoflavone composition in soybeans of different seed coat colors [33]; polyphenol content and antioxidant properties of colored soybean seeds [14,27], and isoflavones, anthocyanins, phenolic content, and antioxidant activities of black soybeans [15,34,35]. However, comprehensive studies on the comparative studies of various components in a large number of soybean genotypes are lacking. The objective of this study was to investigate the protein, amino acids, oil, fatty acids, sugar, anthocyanin, isoflavone, lutein, and antioxidant variations in black, brown, and green seed-coated soybeans.

## 2. Results

### 2.1. Characteristics of Soybean Genotypes

The soybean genotypes included in this study varied in seed coat color, cotyledon color, 100-seed weight, and maturity duration. The soybeans were of black, brown, and green seed coat with green or yellow cotyledons. The 100-seed weight and maturity duration greatly varied from 8.8 g to 66.4 g and from 102 to 155 days, respectively (Table 1).

### 2.2. Protein and Amino Acids

**Figure 1:**
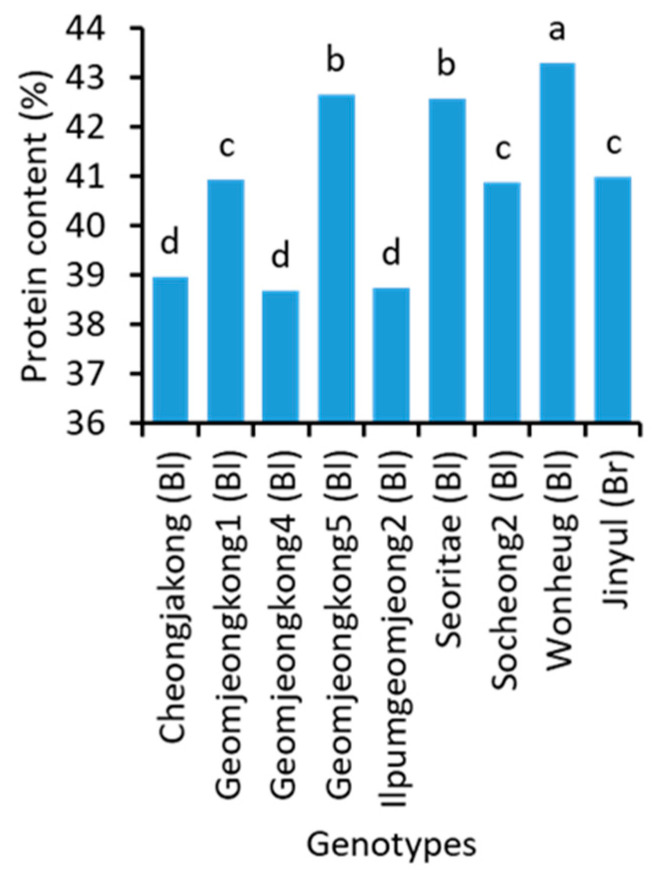
Protein content in the seeds of 9 representative soybean genotypes with high, medium, and low concentrations. Bl and Br, in the parentheses, after the name of genotypes indicate their seed coat color of black and brown, respectively. Different letters above the bars indicate significantly different at *p* < 0.05 (n = 3).

There were significant variations in the protein (Figure 1 and Supplementary Table S1) and amino acid content (Supplementary Table S2) of soybean genotypes. Wonheug (43.3%) had the highest protein content followed by Geomjeongkong5 (42.7%) and Seoritae (42.6%). The lowest (38.7%) protein content was found in Geomjeongkong4 and Ilpumgeomjeong2. The brown and green soybeans had an intermediate level of protein content.

Arginine was significantly high in Seoritae. Tawonkong contained a high amount of the majority of amino acids. Although the amount of a few amino acids found in Tawonkong was significantly equal with those of Taecheong and Wonheug, almost all amino acids were abundantly found in Tawonkong. On the contrary, Cheongyeob1 had the lowest amount of the majority of the amino acids measured in this study.

### 2.3. Oil Content and Fatty Acid Composition

**Figure 2:**
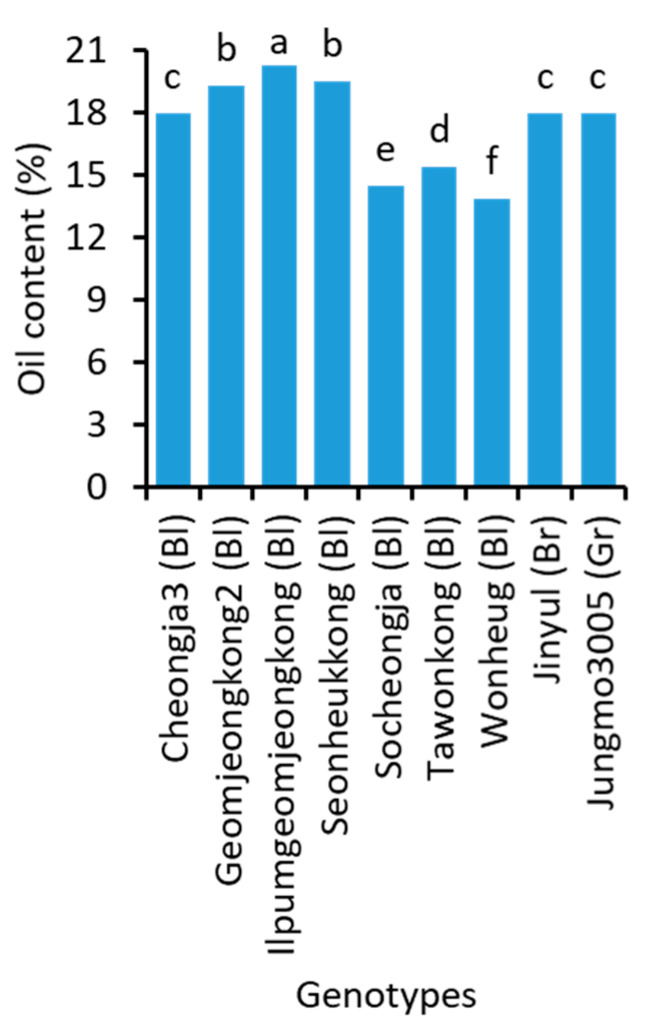
Oil content in the seeds of 9 representative soybean genotypes with high, medium, and low concentrations. Bl, Br, and Gr, in the parentheses, after the name of genotypes indicate their seed coat color of black, brown, and green, respectively. Different letters above the bars indicate significantly different at *p* < 0.05 (n = 2).

The oil (Figure 2 and Supplementary Table S1) and individual fatty acid content (Supplementary Table S3) of soybean genotypes significantly varied. Ilpumgeomjeong2, which had the lowest protein content, showed the highest oil content (20.2%). Similarly, the lowest oil content (13.9%) was found in the highest protein-contained Wonheug. The brown soybean Jinyul and a green soybean Jungmo3005 had significantly equal (17.9%) oil content. 

The highest oil-containing Ilpumgeomjeong2 had the lowest concentration of palmitic (9.7% of oil) and stearic (2.6% of oil) acids. Cheongyeob1, which had the lowest amount of the majority of the amino acids, contained the highest amount of palmitic acid (12.85% of oil). The highest amount of oil-contained Wonheug had the highest stearic acid and lowest oleic acid content. The highest concentration of oleic, linoleic, and linolenic acids were found in Seonheukkong, Socheong2, and Jungmo3009, respectively. Wonheug (10.5% of oil) contained a significantly equal concentration of linolenic acid to that of Jungmo3009.

Positive correlations of stearic acid with palmitic, linoleic, and linolenic acids as well as that of linoleic acid with palmitic and linolenic acids were observed. On the other hand, negative correlations of oleic acid with palmitic, stearic, linoleic, and linolenic acids were found in the present study (Table 2).

### 2.4. Sugar Content

**Figure 3:**
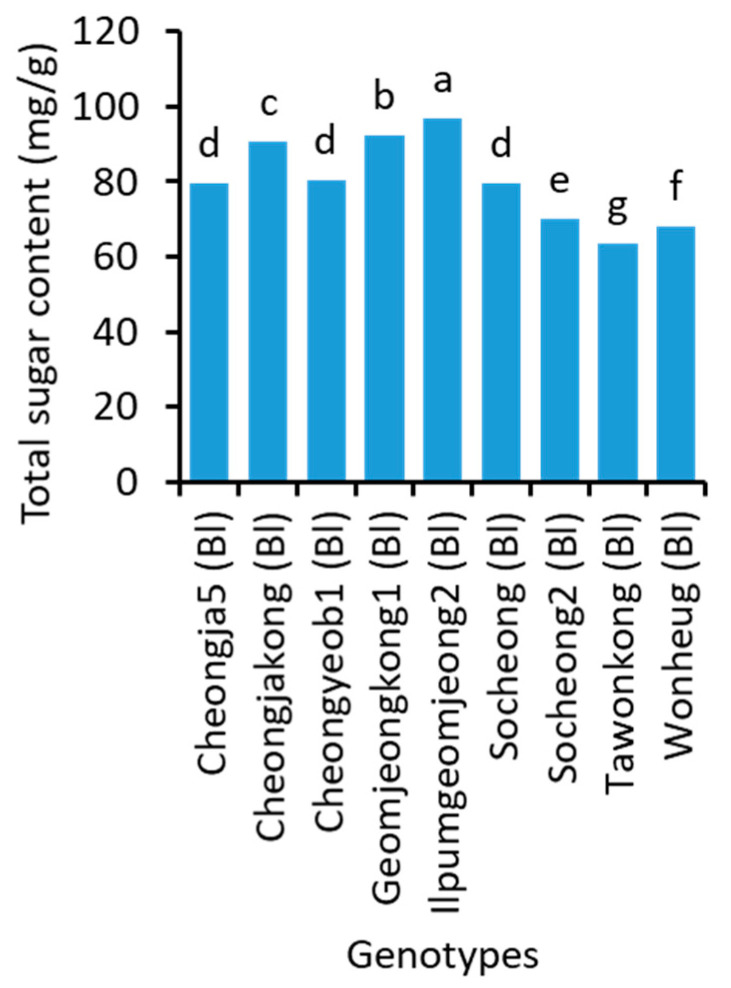
Total sugar content in the seeds of 9 representative soybean genotypes with high, medium, and low concentrations over two years. Bl in the parentheses, after the name of genotypes indicate their black seed coat. Different letters above the bars indicate significantly different at *p* < 0.05 (n = 3).

The total sugar content significantly varied with genotypes (Figure 3 and Supplementary Table S1). Ilpumgeomjeong2, which had the lowest protein and highest oil content, had the highest total sugar content (97.0 mg/g). Tawonkong contained the lowest total sugar (63.5 mg/g), followed by Wonheug (68.1 mg/g) that had the lowest oil and highest protein content. The brown soybean Jinyul (82.3 mg/g) had higher total sugar than two green soybeans Chungdu1 (78.4 mg/g) and Jungmo3005 (74.1 mg/g). The total sugar content had negative correlation with protein, oil, and fructose but positive correlation with stachyose, raffinose, sucrose, glucose, and galactose (Table 3).

The free sugar components also significantly varied among genotypes (Supplementary Table S4). Daeheug had the significantly highest concentration of glucose (6.0 mg/g) and fructose (2.7 mg/g) but had the lowest galactose (1.4 mg/g) content. The highest stachyose (28.2 mg/g) and sucrose (54.6 mg/g) contents were found in Ilpumgeomjeong2. Taecheong contained the highest concentration of raffinose (9.3 mg/g) and galactose (2.5 mg/g). Jungmo3011 (9.3 mg/g) had a significantly equal concentration of raffinose to that of Taecheong.

### 2.5. Isoflavone Content

**Figure 4:**
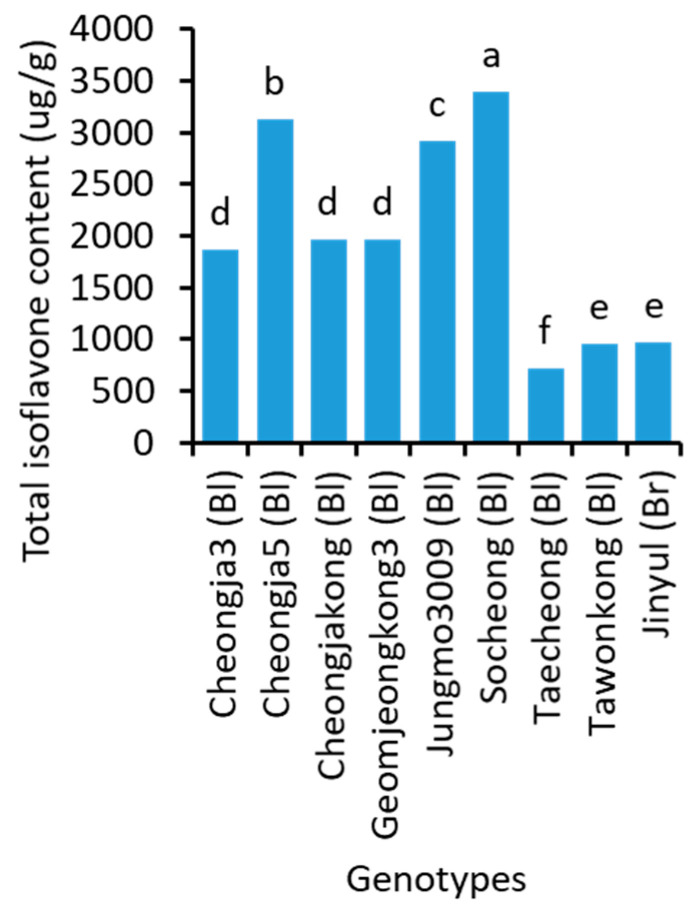
Total isoflavone content in the seeds of 9 representative soybean genotypes with high, medium, and low concentrations. Bl and Br, in the parentheses, after the name of genotypes indicate their seed coat color of black and brown, respectively. Different letters above the bars indicate significantly different at *p* < 0.05 (n = 3).

Significant variations for total isoflavone (Figure 4 and Supplementary Table S1) and individual isoflavone components (Supplementary Table S5) were found among soybean genotypes. Socheong (3394.3 μg/g) had the highest total isoflavone content, followed by Cheongja5 (3132.7 μg/g) and Jungmo3009 (2923.9 μg/g). Taecheong (709.5 μg/g) showed the lowest total isoflavone concentration among the 29 soybeans. The concentration of the total isoflavone in Taecheong was almost 5-fold lesser than that found in Socheong.

Ten black soybeans and one green soybean contained all 12 isoflavones. The other six black soybeans and one green soybean had 11 isoflavones. Out of the 12 isoflavones, a maximum of three individual isoflavones was not detected in six black and one brown soybeans. Six isoflavones: daidzin, glycitin, genistin, malonyldaidzin, malonylglycitin, and malonylgenistin were detected in all 29 genotypes. Acetyldaidzin and acetylglycitin were absent in 12 genotypes. Socheong was the genotype that contained the highest amount of the largest number of isoflavones viz., daidzin (197.8 μg/g), glycitin (63.8 μg/g), malonyldaidzin (1404.5 μg/g), acetyldaidzin (27.2 μg/g), and daidzein (253.6 μg/g). Cheongja5 had significantly equal concentrations of daidzin (192.2 μg/g) and malonyldaidzin (1282.5 μg/g) to that of Socheong. Socheong2 had the highest concentrations of two isoflavones: malonylglycitin (212.4 μg/g) and glycitein (63.1 μg/g). Wonheug had a significantly equal concentration of glycitein to that of Socheong2. Seonheukkong contained the highest concentration of acetylglycitin (23.1 μg/g) and lowest concentrations of daidzin (27.7 μg/g) and malonyldaidzin (211.7 μg/g). Taecheong had the lowest concentrations of three isoflavones: genistein, glycitein, and malonylgenistin. Jungmo3011 also contained a significantly lowest concentration of genistein as Taecheong.

### 2.6. Anthocyanin Content

**Figure 5:**
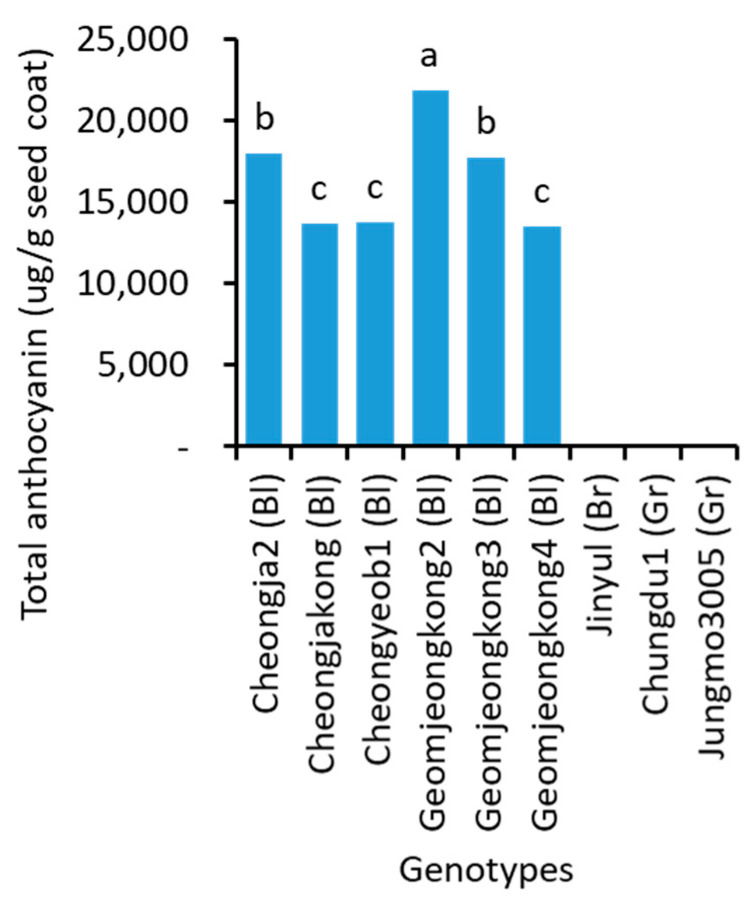
Total anthocyanin content in the seeds of 9 representative soybean genotypes with high, medium, and low concentrations. Bl, Br, and Gr, in the parentheses, after the name of genotypes indicate their seed coat color of black, brown, and green, respectively. Different letters above the bars indicate significantly different at *p* < 0.05 (n = 3).

Like other metabolites, total anthocyanin (Figure 5 and Supplementary Table S1) and individual anthocyanins (Supplementary Table S6) were also significantly different among soybean genotypes. Geomjeongkong2 (21,856 μg/g seed coat) contained the highest total anthocyanin, followed by Cheongja2 (17,953 μg/g seed coat) and Geomjeongkong3 (17,732 μg/g seed coat). Geomjeongkong1 (3826 μg/g seed coat) had the least total anthocyanin content, followed by Tawonkong (5979 μg/g seed coat) and Seoritae (6470 μg/g seed coat). Geomjeongkong2 had almost 6-fold higher total anthocyanin content than that of Geomjeongkong1.

Out of the 29 genotypes, 17 black soybeans contained all 6 anthocyanins measured in the present study. The other 6, 2, and 1 black soybeans had 5, 4, and 3 anthocyanins, respectively. Interestingly, the brown and green soybeans did not show any traces of anthocyanin. Three anthocyanins: cyanidin-3-galactoside, cyanidin-3-glucoside, and peonidin–3-glucoside were detected in all 26 black soybeans. Geomjeongkong3 (3145.1 μg/g seed coat), followed by Cheongja2 (3015.8 μg/g seed coat), contained the highest concentration of delphinidin-3-glucoside. The highest and lowest concentrations of cyanidin-3-galactoside were found in Geomjeongkong2 (273.1 μg/g seed coat) and Geomjeongkong1 (114.2 μg/g seed coat), respectively. Similarly, cyanidin-3-glucoside concentration was also the highest and lowest in Geomjeongkong2 (17,414 μg/g seed coat) and Geomjeongkong1 (3318 μg/g seed coat), although Tawonkong (3063 μg/g seed coat) also had a significantly equal concentration to that of Geomjeongkong1. With regard to the individual anthocyanins, Geomjeongkong1 and Geomjeongkong2 contained the lowest and highest concentrations of the majority of anthocyanins as the total anthocyanin.

### 2.7. Lutein Content

**Figure 6:**
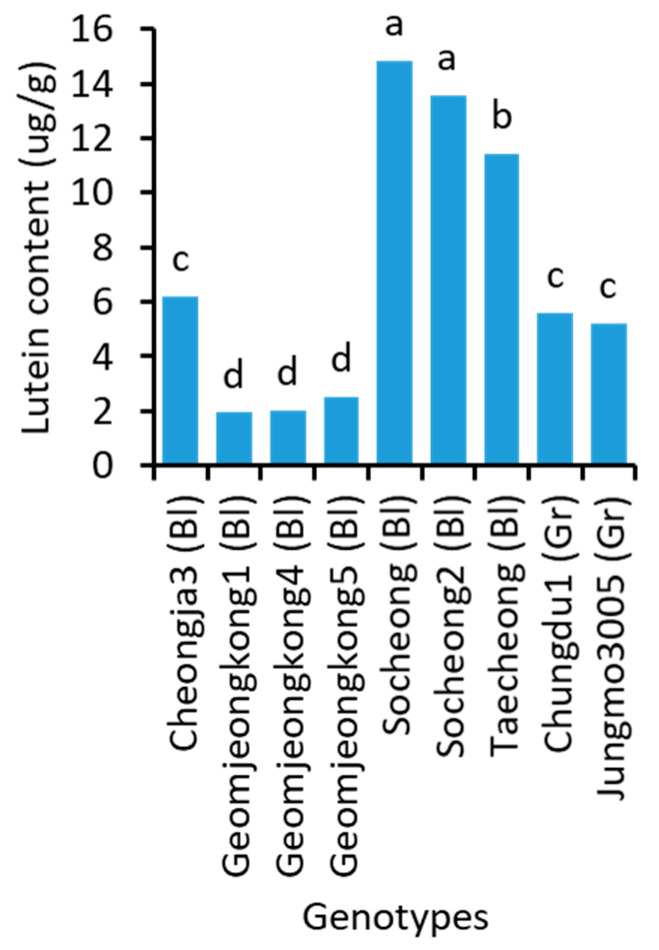
Lutein content in the seeds of 9 representative soybean genotypes with high, medium, and low concentrations. Bl and Gr, in the parentheses, after the name of genotypes indicate their seed coat color of black and green, respectively. Different letters above the bars indicate significantly different at *p* < 0.05 (n = 3).

The lutein content of soybean genotypes significantly varied (Figure 6 and Supplementary Table S1). Cultivar Socheong, which had the highest total isoflavone content, also contained the highest concentration of lutein (14.8 μg/g). The lowest concentration of lutein was detected in Geomjeongkong1 (1.9 μg/g), which also had the lowest total anthocyanin concentration. Lutein was one of the nutrients with high variations among the genotypes (7.6-fold difference between the highest and lowest-containing cultivars). The soybeans with green cotyledons were found to have comparatively higher lutein content than those with yellow cotyledons.

### 2.8. Antioxidants’ Potential

**Figure 7:**
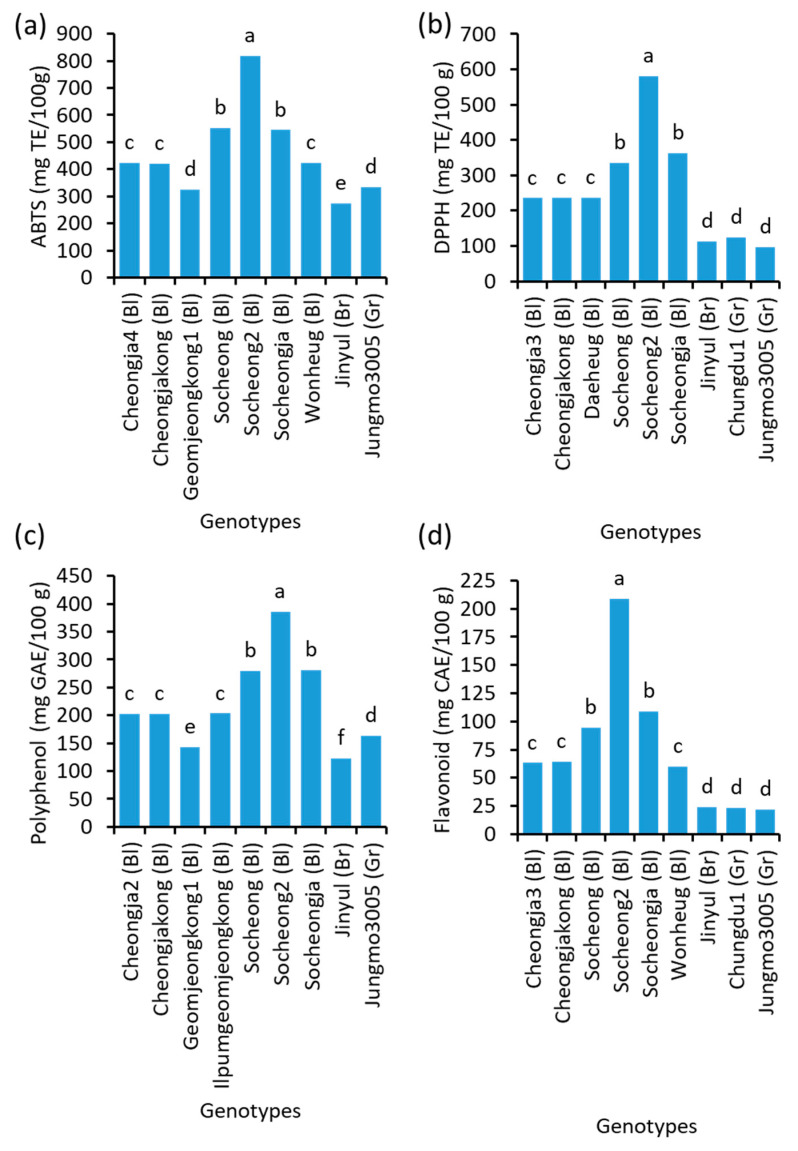
(**a**) ABTS (2,2′-azino-bis(3-ethylbenzthiazoline-6-sulphonic acid) and (**b**) DPPH (2,2-diphenyl-1-picrylhydrazyl) radical scavenging activity, and (**c**) total polyphenol and (**d**) total flavonoid contents in the seeds of 9 representative soybean genotypes with high, medium, and low concentrations. Bl, Br, and Gr, in the parentheses, after the name of genotypes indicate their seed coat color of black, brown, and green, respectively. TE: Trolox equivalent. GAE: gallic acid equivalent. CAE: catechin equivalent. Different letters above the bars indicate significantly different at *p* < 0.05 (n = 4).

The antioxidant potentials of 29 soybeans were determined through ABTS and DPPH free radical scavenging potential and the total polyphenol and total flavonoid contents. These four parameters of antioxidants significantly varied among genotypes (Figure 7 and Supplementary Table S1). A black soybean Socheong2 was found to be the most outstanding genotype with the highest antioxidant potential. The values of ABTS, DPPH, total polyphenol, and total flavonoid of Socheong2 were 818.8 mg Trolox/100 g, 579.7 mg Trolox/100 g, 385.8 mg gallic acid/100 g, and 208.5 mg catechin/100 g, respectively. These values were approximately 3 times higher than that found in a brown soybean Jinjyul for the ABTS (274.9 mg Trolox/100 g) and total polyphenol (123.0 mg gallic acid/100 g), and 6 and 9 times higher for DPPH (96.3 mg Trolox/100 g) and total flavonoid (22.1 mg catechin/100 g) found in a green soybean Jungmo3005. There were highly significant (*p* < 0.0001) linear correlations (as high as r = 0.962 between ABTS and DPPH) between variables of antioxidant potential (Table 4).

## 3. Discussion

The results of protein content were in agreement with previous reports. Dhungana et al. [36] found the seed protein content in the range of 39.4−45.7% in the black soybeans grown in Korea. The protein content varied between 32.2−42.1% in Indian soybeans [37] and 31.8−49.8% in Chinese soybeans [38]. Similarly, despite slight variations in the content of some of the amino acids found in the soybeans grown in the United States [39,40], the concentrations of amino acids found in the present study were within the range of a previous study conducted in different climatic conditions of Argentina [41]. 

The range of oil content was within that found in the black soybeans of Korea (13.45−20.38%) [14]. Slight variations were found in other studies viz., 16.8−24.9% [36], 15.7−17.2% [42], 15.4−22.0% [37], and 14.2−22.8% [38]. The highest and lowest oil contents in the genotypes of lowest and highest protein content might be due to a negative correlation between protein and oil content in soybean [36,38,43]. The ranges of concentration of palmitic, stearic, oleic, linoleic, and linolenic acids were very close to that found in previous studies of soybean cultivars grown in Korea [44,45]. The dominant fatty acid was linoleic acid followed by oleic acid, as found in previous reports [42,46,47]. Inverse relations between oil content and palmitic acid in Ilpumgeomjeong2, between stearic and oleic acids in Wonheug, and between oleic and linoleic acids in Seonheukkong found in the present study were in agreement with a previous report [44]. 

The positive correlation between palmitic and stearic, linoleic and stearic, linolenic and stearic acids as well as the inverse associations of oleic with palmitic, linoleic, and linolenic acids (Table 2) were in agreement with previous results [42,46]. Likewise, the positive associations of linoleic with palmitic and linolenic acids as well as a negative correlation between oleic and stearic acids were different from that found by Abdelghany et al. [46]. The major emphasis of soybean breeding for fatty acids is to enhance the oleic and linoleic acid contents and to decrease linolenic acid content because the latter causes off-flavor and decreases the shelf life of oil [48]. In this context, genotypes like Seonheukkong and Socheong2 could be the potential genotypes for fatty acid breeding. 

Although not significant in all cases, the correlations among different metabolites (Table 3) found in the present study were in agreement with some previous studies but also differed in some cases. The negative correlation between protein and oil content was in agreement with several studies [36,49,50]. The negative correlation of protein with sucrose and total sugar, negative correlation between oil and stachyose, negative correlation between raffinose and stachyose, and positive correlation between oil and sucrose were in agreement with the results of Hymowitz et al. [50]. Additionally, the positive correlation of sucrose with stachyose and raffinose, positive association of total sugar with stachyose, raffinose, and sucrose confirmed the results of Jiang et al. [51]. However, the negative correlation between protein and stachyose, positive association between protein and raffinose, negative relationship between oil and raffinose, and negative correlation between oil and total sugar found in the present study were different from that found by Hymowitz et al. [50]. The disparity might be owing to the extremely high or low concentration of some of the metabolites and/or variations in seed coat color and seed weight of the genotypes [42,52]. 

The isoflavone concentration found in the present study was comparable with several reports. The range of total isoflavone was within that noted in a previous study (682.4–4778.1 μg/g) conducted using the soybeans of different seed sizes from America, China, and Korea [53]. The highest amount of total isoflavone found in the present study was between the range found in the reports of Tepavčević et al. [54] and Xu and Chang [55], however, the lowest amount was slightly lesser than that found by Kumar et al. [56] but comparable (~704 μg/g) with that found in Korean soybeans [57]. Wu et al. [58] found 2276–7258 μg/g total isoflavone in 16 black soybeans grown in China. The total isoflavone ranged from 1450–4590 μg/g in 20 soybeans originating from the United States, Russia, Serbia, and China [54]. A range of 2330–3150 μg/g total isoflavone was also reported in black soybeans [34]. These reports indicate that the isoflavone contents even in black soybeans vary widely. Such disparities might be owing to differences in cultivars, environment, and analysis protocols [59]. With respect to individual isoflavones, malonyldaidzin and malonylgenistin were the most abundant while acetylglycitin and acetylgenistin were the least abundant isoflavones in the present study. These results were analogous with a previous study for high malonylgenistin [13,53] and malonyldaidzin [34,58]. Although the concentrations were wide-ranging, acetylglycitin and acetylgenistin were found as the least present isoflavones in the previous studies [34,54] as well. 

The range of total anthocyanins found in the present study was within that (1894.6–26,334.5 μg/g) found in a previous report [35]. They also noted that the variation in anthocyanin content was influenced by soybean seed size along with seed coat color. Another study [60] with a greater variation (988–21,325 μg/g) of total anthocyanin content has also been reported. Many soybean cultivars with brown and green seed coats were not found to contain anthocyanins [14]. As far as the individual anthocyanins are concerned, cyanidin-3-galactoside accounted for the most abundant anthocyanin among others. A higher cyanidin-3-galactoside was also found in previous reports [35,53,60]. Delphinidin-3-glucoside and petunidin-3-glucoside were detected as the other two major anthocyanins in terms of their share in total anthocyanins which was in agreement with the results of earlier studies [13,14,15,35]. The range of individual anthocyanins found in the present study was comparable with that found in the report of Zhang et al. [60] for delphinidin-3-glucoside (0–3949), cyanidin-3-galactoside (0–439 μg/g), cyanidin-3-glucoside (618–16,174 μg/g), petunidin-3-glucoside (38–1982 μg/g), and peonidin-3-glucoside (3–1760 μg/g) with some variation for cyanidin-3-glucoside. Several factors such as cultivars, growing conditions, and season may account for such differences in individual anthocyanin contribution to total anthocyanins [55,60].

The results of higher lutein contents in the soybeans with green cotyledons were in agreement with previous reports [61,62]. The lutein content found in the present study was comparable with that found in previous reports viz., 0.39–8.61 μg/g [63], 1.32–13.95 μg/g [61], and 4.1–10.9 μg/g [64]. The lutein content of wild soybeans (5.8−32.8 μg/g) was higher than that of cultivated soybeans (1.6–14.8 μg/g) [62]. Slight variations in the composition of soybeans can be expected owing to differences in genotypes, farming conditions, and environments [65,66].

The significant linear correlations (Table 4) between different variables of antioxidants found in the present study were similar to those observed in previous studies. Positive correlation between phenolic contents and DPPH [35,60], DPPH and ABTS [14], and ABTS and total phenolic [18]. Phenolic compounds such as flavonoids (especially isoflavones) and anthocyanins found in soybeans serve as antioxidants [13,27]. The high antioxidant activities and significant correlation among different variables might be owing to the availability of different phytochemicals in the soybean genotypes considered in the present study.

## 4. Materials and Methods

### 4.1. Plant Materials and Growing Conditions

A total of 29 (26 black, 1 brown, and 2 green) colored seed-coated Korean soybean genotypes were considered in this study (Table 1). 

The nutrient content and antioxidant potentials were evaluated in the soybeans grown in the fields of the Department of Southern Area Crop Science Miryang (35°29′32″ N 128°44′35″ E), Korea in 2018 and 2019. The experiments were performed in a randomized complete block design with three replications. Seeds were sown at the spacing of 70 cm between rows and 15 cm between plants. Plants were grown according to the cultivation methods of Agricultural Science Technology Standards for Investigation (Rural Development Administration, Jeonju, Korea). The plants were harvested at maturity and dried seeds were used for analysis.

### 4.2. Chemicals and Reagents

Lutein, 2,2′-azino-bis(3-ethylbenzthiazoline-6-sulphonic acid) (ABTS), 2,2-diphenyl-1-pycrylhydrazyl (DPPH), 6-hydroxy-2,5,7,8-tetramethylchroman-2-carboxylic acid (Trolox), (+)-catechin, gallic acid, trifluoroacetic acid, Folin–Ciocalteu phenol reagent, sodium carbonate, sodium nitrite, and sodium hydroxide were purchased from Sigma-Aldrich Chemical Co. (St. Louis, MO, USA). The HPLC grade water, ethanol, and methanol were obtained from J.T. Baker (Phillipsburg, NJ, USA). All other chemicals used in the present study were of analytical grade.

### 4.3. Protein and Amino Acids’ Analysis

The protein content was determined using an Elementar Rapid N Cube (Hanau, Germany) following the manufacturer’s instructions. Fifty milligrams of seed powder were put in parchment paper-making pellets that were put into the machine. The temperatures of the combustion tube, post-combustion tube, and reduction tube were 955, 750, and 830 °C, respectively. The protein content was detected at 59.8 °C.

The amino acid content in soybean seeds was measured using an amino acid analyzer (Biochrom 30, Biochrom Ltd., Cambridge, UK) following the method described by Je et al. [67]. Each sample containing 100 mg seed powder was hydrolyzed with 3 mL of 6 M hydrochloric acid at 110 °C for 24 h. The hydrolysate was filtered into another tube and brought to a known volume of 5 mL with 0.2 M citrate buffer (pH 3.2). Ninhydrin as a color reactant and sodium column (80-2104-15) were used to determine amino acids. 

For the determination of free amino acids, 0.5 mg seed powder was extracted with 1 mL distilled water for 2 h using ultrasonication. The mixture was centrifuged at 2000× *g* for 30 min and the supernatant was filtered through a Whatman No. 41 filter paper. The filtrate was diluted 10 times with lithium buffer (pH 2.8). The diluted filtrates were then analyzed on the same amino acid analyzer using a lithium column (80-6002-49).

### 4.4. Measurement of Oil and Fatty Acids

The oil content of soybean seeds was measured by the Soxhlet method [5] using a thimble (Buchi Extraction Thimbles 25 × 100 mm) extraction system (Buchi B-811, Büchi, Switzerland). Two grams of the powdered seeds were put into the extraction thimbles and added with *n*-hexane. The system was run at 105 °C for 2 h 40 min and cooled to room temperature in a desiccator, and then the weight of extracted oil was measured and expressed on a dry mass basis.

The fatty acid profile of the extracted oil was determined using a gas chromatography (Agilent 7890A series, Boeblingen, Germany) method using a solvent (methanol: toluene: sulfuric acid: 20: 10: 1, *v/v/v*). Five milliliters of the solvent were mixed with 200 μL oil and kept in a dryer (105 °C) for 1 h. Then, 1 mL distilled water was added to the mixture and mixed thoroughly. The injecting extract for GC analysis was prepared by taking the supernatant into a 1.5 mL tube and mixing a small amount of Na_2_SO_4_. One microliter extract was injected onto the gas chromatography column in a split mode. An Agilent 19091F-413 capillary column (30 m × 320 μm, 0.25 μm, Agilent, Santa Clara, CA, USA) and a flame ionization detector were used. The initial temperature of 140°C was increased to the final 200 °C temperature at a rate of 8 °C/min. Nitrogen was used as the carrier gas. Individual fatty acid standards were calibrated and the amounts of fatty acids in the sample extracts were measured by comparing the gas chromatography retention times with those of the standards. The concentrations of individual fatty acids were determined as the percentage of the oil content.

### 4.5. Determination of Free Sugar Content

The free sugar content was analyzed using HPLC (Ultimate 3000 HPLC, Dionex, Sunnyvale, CA, USA) system following the method described by Kim et al. [68] with some modifications. One gram of seed powder was extracted with 10 mL 70% ethanol by continuous stirring at room temperature for 3 h, followed by 24 h of refrigeration, and then centrifugation at 13,500 rpm for 10 min. The supernatant was diluted with an equal volume of distilled water, passed through a 0.2 μm filter, and collected in 1.5 mL vials for HPLC analysis. The operating conditions were as follows—column: Sugar Pak (Waters, 6.5 mm × 0.32 mm), solvent: distilled water, flow rate: 0.5 mL/min, detector: refractive index detector (Shodex RI, Tokyo, Japan), and column temperature: 80°C. The free sugar contents were calculated from the standard calibration curves. 

### 4.6. Isoflavone Analysis

One gram of seed powder was extracted with 20 mL of 50% methanol by continuous stirring at room temperature for 24 h, followed by centrifugation (13,500 rpm, 10 min). The supernatant was collected in 1.5 mL vials after passing through a 0.2 μm filter. The isoflavone content was determined using an HPLC system (Ultimate 3000 HPLC, Dionex, Sunnyvale, CA, USA) following the method described by Lee et al. [69] with some modifications. The operating conditions were as follows—column: Lichrospher RPC18 (5 μm, 4 mm × 125 mm), solvent A: distilled water with 0.1% acetic acid, solvent B: acetonitrile with 0.1% acetic acid, flow rate: 1 mL/min, detector: UV-vis detector, sample injection amount: 10 μL.

### 4.7. Anthocyanin Analysis

The anthocyanin content of soybean seed coats was analyzed by following the method described by Lee et al. [6]. Hand-peeled seed coats (0.1 g) were extracted with 30 mL of 20% methanol containing 1% (*v/v*) hydrochloric acid for 48 h under refrigerated conditions. The mixture was centrifuged (3000× *g*, 3 min) at room temperature and the supernatant was filtered using a 0.2 μm filter. The anthocyanin content was determined using an HPCL system (Ultimate 3000 HPLC, Dionex, Sunnyvale, CA, USA) with a flow rate of 0.8 mL/min. The HPLC operating conditions were as follows—column: YMC-Triart C18 (4.6 × 150 mm, 5 μm), solvent A: 0.1% trifluoroacetic acid in distilled water, solvent B: 0.1% trifluoroacetic acid in methanol, detector: UV-vis detector (530 nm), and analysis time: 45 min.

### 4.8. Determination of Lutein Content

The lutein content was determined using an HPLC system (Ultimate 3000 HPLC, Dionex) following the method described by Seguin et al. [64] with some modification. Sample extracts were prepared with acetone (20 mL) from 1 g seed powder by ultrasonic extraction for 30 min. After centrifugation (3000× *g*, 3 min) at room temperature, the supernatant was passed through a 0.2 μm filter. The HPLC was run at the flow rate of 1 mL/min. The other conditions for HPLC analysis were as follows—column: Supersil (Dalian, China, 10 nm, 5 μm, 4.6 × 150 mm), solvent A: 75% methanol, solvent B: ethyl acetate, detector: UV-vis (450 nm), injection volume: 10 μL. 

### 4.9. Sample Extraction for ABTS, DPPH, Total Polyphenol, and Total Flavonoid Analyses

Twenty grams of soybean seeds were ground into powder using a commercial grinder. Two grams of powder was extracted with 10 mL 80% ethanol for 24 h using a shaking incubator (240 rpm). The extraction was repeated with a fresh 10 mL ethanol after transferring the old extract into a new falcon tube. The first and second extracts were mixed and the mixture was centrifuged (3000× *g*) at room temperature for 3 min and the supernatant was filtered through a 0.45 μm syringe filter.

### 4.10. ABTS Radical Scavenging Assay

The ABTS radical scavenging effect was determined following the method described by Lee and Cho [5] with slight modifications. The 7.4 mM ABTS^+^ and 2.6 mM potassium persulphate solutions prepared in ethanol were mixed at a 1:1 ratio and left in the dark at room temperature for about 14 h to make an ABTS^+^ stock solution. The stock solution was diluted with ethanol to get an absorbance value of 0.2–1.0 at 735 nm. Two hundred microliters of stock solution and 20 μL sample extracts and Trolox as the standard (Supplementary Figure S1) were put into a 96-well plate. The reaction mixture was allowed to react for 30 min and then the absorbance was measured at 735 nm using a spectrophotometer (Thermo, Multiskan Spectrum, Vantaa, Finland). The DPPH radical scavenging potential was expressed as Trolox equivalent.

### 4.11. DPPH Radical Scavenging Assay

The DPPH radical scavenging activity was measured according to a previous method [14]. Twenty microliters of sample extracts and Trolox as the standard (Supplementary Figure S2) were put into a 96-well plate. Two hundred microliters of 0.2 mM DPPH solution were added to the wells and mixed by pipetting. The reaction mixture was placed in the dark for 30 min and then the absorbance of the mixture was measured at 520 nm using a spectrophotometer (Thermo, Multiskan Spectrum). The DPPH radical scavenging potential was calculated as Trolox equivalent.

### 4.12. Measurement of Total Polyphenol Content

The total polyphenol content was evaluated according to the Folin–Ciocalteu method as described by Celli et al. [70] with a slight modification. Sample extracts (20 μL) and 10% Folin–Ciocalteu reagent (100 μL) were mixed in a 96-well plate and allowed to react for 5 min, followed by adding 80 μL of 7.5% Na_2_CO_3_ and incubating in the dark for 30 min. Gallic acid (GA) was used to plot the standard calibration curve (Supplementary Figure S3), for which the sample extract was replaced with 0, 50, 100, 250, and 500 ppm concentrations of GA in the above procedure. The absorbance values of the reaction mixtures were read at 750 nm using a spectrophotometer (Thermo, Multiskan Spectrum).

### 4.13. Measurement of Total Flavonoid Content

The total flavonoid content was determined by following the method described by Celli et al. [70] with some modifications. A mixture of 100 μL sample extracts, 400 μL distilled water, and 30 μL 5% NaNO_2_ was vortexed in 1.5 mL tubes and left for 5 min, followed by mixing 30 μL 10% AlCl_3_ and leaving for 6 min. After 6 min, 200 μL of 1 M NaOH and 240 μL distilled water were added to the mixture and vortexed. The absorbance of the reaction mixture was measured at 510 nm using a spectrophotometer (Thermo, Multiskan Spectrum). The calibration curve (Supplementary Figure S4) was plotted using catechin hydrate and the total flavonoid content of the sample was expressed as catechin equivalent.

### 4.14. Statistical Analysis

The data were analyzed with analysis of variance using SAS9.4 (SAS Institute, Cary, NC, USA) to compare the means of different genotypes. The significant differences among genotypes were determined at *p* < 0.05 level using the least significant difference test. The average values of two to four measurements were considered for statistical analysis. The correlation coefficient among different components was measured using the PROC CORR function in SAS9.4.

## 5. Conclusions

The protein, amino acids, oil, fatty acids, sugars, anthocyanins, isoflavones, lutein, total polyphenol, and total flavonoid contents as well as ABTS and DPPH-radical scavenging potentials of 26 black, 2 brown, and 1 green seed-coated soybeans were determined. Both contents and antioxidant potentials were significantly different among the studied genotypes. Ilpumgeomjeong2 had the lowest protein but the highest oil and total sugar contents. The lowest oil-containing soybean, Wonheug, was found to have the highest protein content. The correlations among protein, oil, and sugar contents were in agreement with previous studies. Similarly, strong correlations among antioxidant activities (ABTS and DPPH) and total polyphenol and total flavonoid contents were found. A black soybean, Socheong2, had consistently high values of these four variables of antioxidants. Black soybeans had relatively higher antioxidant potentials than brown and green soybeans. Anthocyanins were detected only in black soybeans but not in brown and green soybeans. The variation in isoflavone content was up to 5-fold among the soybean genotypes. The results of this study could provide a piece of valuable information for the quality improvement of soybeans because an understanding of the nutrient content and antioxidant potentials of soybean genotypes of different seed coat colors is useful to prioritize the effective strategies for improving the economic traits of soybeans.

## Figures and Tables

**Table 1:** Name, seed coat color, cotyledon color, 100-seed weight, status, and days to maturity of 29 soybean genotypes.

SN	Genotype	Seed Coat Color	Cotyledon Color	HSW (g)	Status	Days to Maturity (Category)
1	Cheongja2	Black	Green	26.0	Cultivar	108 (Early)
2	Cheongja3	Black	Green	32.1	Cultivar	117 (Mid)
3	Cheongja4	Black	Green	30.1	Cultivar	126 (Late)
4	Cheongja5	Black	Green	37.0	Cultivar	131 (Extremely late)
5	Cheongjakong	Black	Green	30.4	Cultivar	111 (Mid)
6	Cheongyeob1	Black	Yellow	35.8	Cultivar	117 Mid
7	Daeheug	Black	Yellow	34.3	Cultivar	108 (Early)
8	Geomjeongkong1	Black	Yellow	29.8	Cultivar	107 (Early)
9	Geomjeongkong2	Black	Yellow	28.3	Cultivar	112 (Mid)
10	Geomjeongkong3	Black	Yellow	31.0	Cultivar	111 (Mid)
11	Geomjeongkong4	Black	Yellow	28.0	Cultivar	107 (Early)
12	Geomjeongkong5	Black	Yellow	23.2	Cultivar	111 (Mid)
13	Heugmi	Black	Yellow	24.8	Cultivar	105 (Early)
14	Heugsung	Black	Yellow	29.2	Cultivar	110 (Early)
15	Ilpumgeomjeong2	Black	Yellow	25.0	Cultivar	109 (Early)
16	Ilpumgeomjeongkong	Black	Yellow	28.0	Cultivar	102 (Early)
17	Jungmo3009	Black	Green	29.3	Cultivar	126 (Late)
18	Jungmo3011	Black	Green	66.4	Cultivar	155 (Extremely Late)
19	Seonheukkong	Black	Yellow	34.2	Cultivar	112 (Mid)
20	Seoritae	Black	Green	40.0	Landrace	148 (Extremely Late)
21	Socheong	Black	Green	15.7	Cultivar	113 (Mid)
22	Socheong2	Black	Green	12.2	Cultivar	109 (Early)
23	Socheongja	Black	Green	12.0	Cultivar	120 (Mid)
24	Taecheong	Black	Green	44.5	Cultivar	127 (Late)
25	Tawonkong	Black	Yellow	9.4	Cultivar	105 (Early)
26	Wonheug	Black	Yellow	8.8	Cultivar	111 (Mid)
27	Jinyul	Brown	Yellow	28.3	Cultivar	107 (Early)
28	Chungdu1	Green	Green	23.6	Cultivar	113 (Mid)
29	Jungmo3005	Green	Green	24.3	Cultivar	114 (Mid)

HSW: Hundred-seed weight. The genotypes were categorized as early (<110 days), mid (111−120 days), late (121−130 days), and extremely late (>131 days) based on the number of days from sowing to maturity.

**Table 2:** Pearson correlation among different fatty acids measured in the seeds of 29 soybean genotypes.

	Palmitic	Stearic	Oleic	Linoleic	Linolenic
Palmitic	1				
Stearic	0.361 **	1			
Oleic	−0.299 *	−0.411 **	1		
Linoleic	0.152	0.347 **	−0.973 ***	1	
Linolenic	0.100	0.077	−0.713 ***	0.595 ***	1

Symbols *, **, and ***, followed by the Pearson correlation coefficient values, indicate statistically significant correlation at *p* < 0.05, 0.01, and 0.0001, respectively.

**Table 3:** Pearson correlation among protein, oil, stachyose, raffinose, sucrose, glucose, galactose, fructose, and total sugar measured in the seeds of 29 soybean genotypes.

	Protein	Oil	Stachyose	Raffinose	Sucrose	Glucose	Galactose	Fructose	Total Sugar
Protein	1								
Oil	−0.525 ****	1							
Stachyose	−0.235 **	−0.151	1						
Raffinose	0.213 **	−0.279 *	−0.103	1					
Sucrose	−0.034	0.016	0.125	0.4367 ****	1				
Glucose	0.132	0.048	−0.487 ****	0.274 ***	0.297 ****	1			
Galactose	−0.126	−0.068	−0.082	0.282 ***	0.088	−0.131	1		
Fructose	−0.106	0.072	−0.399 ***	−0.232	−0.282 **	0.531 ****	0.050	1	
Total sugar	−0.074	−0.036	0.318 ****	0.461 ****	0.967 ****	0.272 ***	0.120	−0.239 *	1

Symbols *, **, ***, and ****, followed by the Pearson correlation coefficient values, indicate statistically significant correlation at *p* < 0.05, 0.01, 0.001, and 0.0001, respectively.

**Table 4:** Pearson correlation among ABTS (2,2′-azino-bis(3-ethylbenzthiazoline-6-sulphonic acid) and DPPH (2,2-diphenyl-1-picrylhydrazyl) radical scavenging activity, and total polyphenol and total flavonoid contents in the seeds of 29 soybean genotypes.

	ABTS	DPPH	Flavonoid	Polyphenol
ABTS	1			
DPPH	0.962 ****	1		
Flavonoid	0.945 ****	0.932 ****	1	
Polyphenol	0.953 ****	0.896 ****	0.924 ****	1

Symbol ****, followed by the Pearson correlation coefficient values, indicates statistically significant correlation at *p* < 0.0001.

## Data Availability

The data generated in this study are included in this published article and its Supplementary materials.

## Supplementary Materials

The following are available online at https://www.mdpi.com/article/10.3390/plants10091765/s1, Figure S1: Standard calibration curve for ABTS (2,2′-azino-bis(3-ethylbenzthiazoline-6-sulphonic acid) radical scavenging assay, Figure S2: Standard calibration curve for DPPH (2,2-diphenyl-1-picrylhydrazyl) radical scavenging assay, Figure S3: Standard calibration curve for total polyphenol measurement, Figure S4: Standard calibration curve for total flavonoid determination, Table S1: Summary of nutrient content and antioxidant potential of 29 soybean genotypes, Table S2: Amino acid concentration (mg/g) in the seeds of 29 soybean genotypes, Table S3: Fatty acid content (% of oil content) in the seeds of 29 soybean genotypes, Table S4: Free sugar concentration (mg/g) in the seeds of 29 soybean genotypes, Table S5: Isoflavone content (μg/g) in the seeds of 29 soybean genotypes, Table S6: Anthocyanin content (μg/g seed coat) in the seeds of 29 soybean genotypes.
