# Supplementary material for: Protein, Amino Acid, Oil, Fatty Acid, Sugar, Anthocyanin, Isoflavone, Lutein, and Antioxidant Variations in Colored Seed-Coated Soybeans
Source: Plants (Basel). 2021 Aug 25;10(9):1765. doi: 10.3390/plants10091765 (PMC8468453; doi:10.3390/plants10091765)
Supplement: Supplementary file 1 [file plants-10-01765-s001.zip › plants-1316171-supplementary.pdf]

Article

Protein, Amino Acid, Oil, Fatty Acid, Sugar, Anthocyanin, Isoflavone, Lutein, and Antioxidant Variations in Colored Seed-Coated Soybeans

Sanjeev Kumar Dhungana, Jeong-Hyun Seo *, Beom-Kyu Kang, Ji-Hee Park, Jun-Hoi Kim, Jung-Sook Sung, In-Youl Baek, Sang-Ouk Shin, Chan-Sik Jung

Upland Crop Breeding Research Division, Department of Southern Area Crop Science, National Institute of Crop Science, Rural Development Administration, Miryang 50424, Republic of Korea; sanjeev@korea.kr (S.K.D), next0501@korea.kr (J.-H.S.), hellobk01@korea.kr (B.-K.K.), heeya91@korea.kr (J.-H.P.), itomi123@korea.kr (J.-H.K.), sjs31@korea.kr (J.-S.S.), baekiy@korea.kr (I.-Y.B.), shinso32@korea.kr (S.-O.S.), jung100@korea.kr (C.-S.J.)

* Correspondence: next0501@korea.kr; Tel.: (+82-55-350-1236)

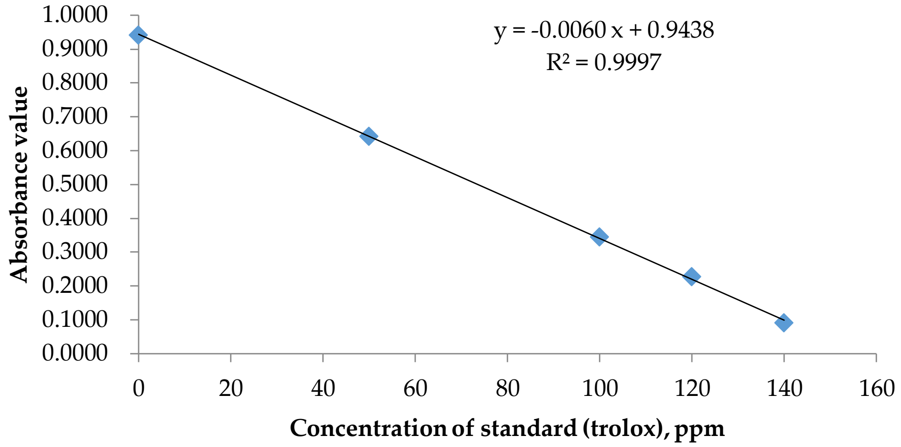

Supplementary Figure S1. Standard calibration curve for ABTS (2,2′-azino-bis(3-ethylbenzthiazoline-6-sulphonic acid) radical scavenging assay.

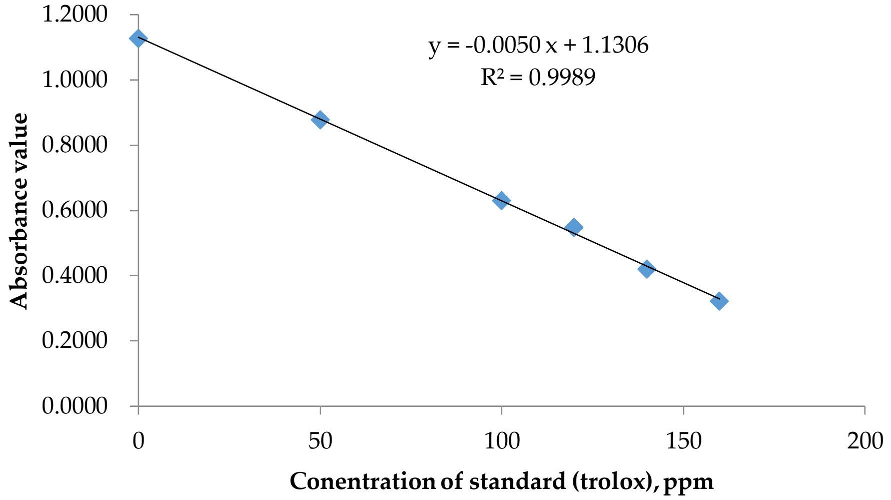

Supplementary Figure S2. Standard calibration curve for DPPH (2,2-diphenyl-1-picrylhydrazyl) radical scavenging assay.

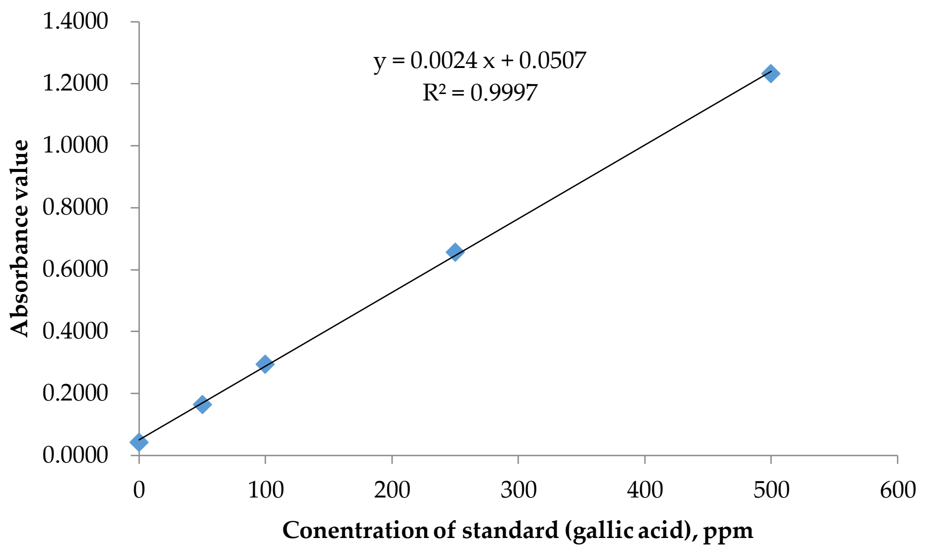

Supplementary Figure S3. Standard calibration curve for total polyphenol measurement.

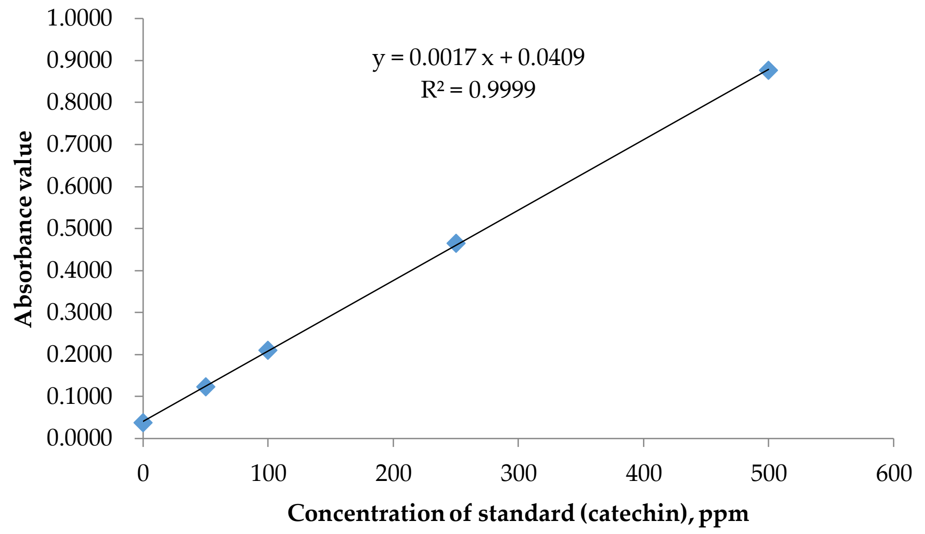

Supplementary Figure S4. Standard calibration curve for total flavonoid determination.

Supplementary Table S1. Summary of nutrient content and antioxidant potential of 29 soybean genotypes.

Genotype	Protein (%)	Oil (%)	Total sugar (mg/g)	Isoflavone (µg/g)	Anthocyanin (µg/g seed coat)	Lutein (µg/g)	ABTS (mg TE/100 g)	DPPH (mg TE/100 g)	Polyphenol (mg GAE/100 g)	Flavonoid (mg CAE/100 g)
Cheongja2 (Bl)	41.4 ef	16.9 m	85.2 e	2410.3 e-h	17953 b	6.5 g-i	411.1 k	205.4 m	203.2 i-k	54.4 lm
Cheongja3 (Bl)	41.0 f-i	18.0 i-k	78.5 l	1868.6 j-l	12164 g-j	6.2 g-j	425.1 hi	235.3 kl	195.5 k	63.2 i
Cheongja4 (Bl)	40.1 kl	18.7 d-g	87.2 d	1866.2 j-l	12499 f-i	11.1 c	422.5 i	248.2 i	206.0 gh	66.0 gh
Cheongja5 (Bl)	40.0 lm	18.8 d-f	79.6 j-l	3132.7 ab	17547 bc	8.7 d-f	500.4 d	291.7 ef	252.2 c	81.3 d
Cheongjakong (Bl)	39.0 op	19.1 b-d	90.6 bc	1965.4 i-l	13648 d-h	6.3 g-i	421.1 ij	237.0 i-k	202.1 i-k	64.5 hi
Cheongyeob1 (Bl)	40.6 i-k	18.4 f-i	80.6 i-k	1610.6 l	13789 d-h	4.4 i-m	428.1 h	262.9 h	207.6 f-h	68.2 g
Daeheug (Bl)	40.8 h-j	19.0 c-e	73.2 o	1741 kl	16829 b-d	3.7 k-n	407.2 k	236.0 i-k	186.0 l	57.1 k
Geomjeongkong1 (Bl)	41.0 f-i	17.8 jk	92.3 b	1153.7 m	3826 l	1.9 n	324.1 p	155.0 n	143.1 q	35.9 o
Geomjeongkong2 (Bl)	39.6 mn	19.3 bc	82.5 gh	2451.5 e-g	21856 a	3.8 k-n	503.1 d	316.2 d	240.8 d	76.4 e
Geomjeongkong3 (Bl)	41.2 e-h	17.6 kl	87.0 d	1968.2 i-l	17732 bc	5.0 h-l	482.7 e	295.6 e	230.8 e	72.5 f
Geomjeongkong4 (Bl)	38.7 p	18.9 c-e	83.2 fg	2860.1 b-d	13473 e-h	2.0 n	439.8 g	232.3 j-l	209.7 fg	58.5 jk
Geomjeongkong5 (Bl)	42.7 b	18.2 g-j	75.7 m	1784.7 j-l	9794 ij	2.5 mn	391.4 l	221.9 l	192.4 k	46.1 n
Heugmi (Bl)	40.3 j-l	19.1 b-d	74.6 m-o	1652.4 l	11196 h-j	8.0 e-g	410.4 k	242.7 ij	181.9 lm	52.0 m
Heugsung (Bl)	42.0 d	16.9 m	84.6 ef	1676.6 l	10144 ij	4.1 j-n	416.8 j	235.0 jk	198.6 i-k	52.5 m
Ilpumgeomjeong2 (Bl)	38.7 p	15.6 o	97.0 a	2581.9 c-f	15593 b-f	3.4 l-n	470.2 f	279.2 fg	229.3 e	67.3 g
Ilpumgeomjeongkong (Bl)	39.4 n	20.2 a	73.9 no	2508.1 d-f	13937 d-h	3.1 l-n	424.3 hi	230.7 j-l	203.3 i-k	52.9 m
Jungmo3009 (Bl)	40.6 ij	16.3 n	89.4 c	2923.9 bc	16666 b-e	9.7 c-e	469.8 f	270.9 gh	240.3 d	74.6 ef
Jungmo3011 (Bl)	41.6 de	17.9 i-k	79.0 kl	1025.2 mn	14700 c-g	6.6 f-h	387.4 l	239.0 i-k	184.7 l	67.0 g
Seonheukkong (Bl)	39.6 mn	19.5 b	75.5 mn	1011.1 mn	11259 h-j	3.1 l-n	381.4 m	227.6 kl	172.5 no	57.1 k
Seoritae (Bl)	42.5 b	17.3 lm	81.3 h-j	2080.6 h-k	6470 kl	9.4 c-e	417.2 j	232.3 j-l	213.8 f	67.3 g
Socheong (Bl)	39.4 no	16.9 m	79.8 j-l	3394.3 a	14602 c-g	14.8 a	552.1 b	335.4 c	279.4 b	94.3 c
Socheong2 (Bl)	40.9 g-i	17.3 lm	70.2 p	2742.3 c-e	16013 b-e	13.5 ab	818.8 a	579.7 a	385.8 a	208.5 a
Socheongja (Bl)	41.3 e-g	14.5 p	74.8 m-o	2116.7 g-j	15173 b-g	10.5 cd	544.7 c	363.0 b	280.6 b	109.1 b
Taecheong (Bl)	42.5 bc	18.2 h-j	83.0 f-h	709.5 n	15507 b-f	11.4 bc	389.1 l	231.9 j-l	175.7 mn	56.8 kl
Tawonkong (Bl)	42.1 cd	15.4 o	63.5 r	955.2 mn	5979 kl	4.0 k-n	425.9 hi	242.0 ij	197.7 jk	67.2 g
Wonheug (Bl)	43.3 a	13.9 q	68.1 q	1785 j-l	9024 jk	6.3 g-i	423.1 hi	230.8 j-l	204.3 g-i	59.8 j
Jinyul (Br)	41.0 f-i	17.9 i-k	82.3 g-i	971.8 mn	ND	3.6 k-n	274.9 q	112.0 o	123.0 r	24.3 p
Chungdu1 (Gr)	41.1 f-h	18.6 e-h	78.4 l	2622.6 c-f	ND	5.6 h-k	355.0 n	124.3 o	167.7 op	23.4 p
Jungmo3005 (Gr)	41.9 d	17.9 i-k	74.1 m-o	2285.0 f-i	ND	5.2 h-l	332.8 o	96.3 p	163.8 p	22.1 p
LSD (5%)	0.45	0.47	1.76	319.70	3241	2.16	5.28	12.57	6.35	2.43

Bl, Br, and Gr, after the name of genotypes indicate their seed coat color black, brown, and green, respectively. TE: Trolox equivalent. GAE: gallic acid equivalent. CAE: catechin equivalent. Mean values followed by different letters in the same column indicate significantly different ($p < 0.05$). ND: non-detectable.

Supplementary Table S2. Amino acid concentration (mg/g) in the seeds of 29 soybean genotypes.

Genotype	Arg	His	Ile	Leu	Lys	Met	Phe	Thre	Val	Ala	Asp	Glu	Ser	Amm	Cys	Gly	Pro	Tyr
Cheongja2 (Bl)	24.6 n	9.1 k	15.4 f-h	27.0 i-k	21.6 lm	3.1 m	17.6 gh	13.2 g-i	16.2 j-l	14.6 h	37.8 kl	68.6 h-j	17.7 i-l	21.0 ij	1.4 g-i	14.7 h-j	16.9 e-i	10.8 gh
Cheongja3 (Bl)	26.7 g-i	9.1 jk	14.9 k	26.9 j-l	21.7 kl	3.5 k	17.3 h-k	12.8 i-k	15.7 mn	14.5 hi	38.2 i-k	67.0 kl	17.6 j-l	21.7 ef	1.1 i	14.3 kl	16.7 f-i	10.5 jk
Cheongja4 (Bl)	28.7 e	8.9 l	14.4 n	25.5 q	20.9 o	3.6 h	16.7 n	12.3 l	15.3 o	14.1 j	37.1 lm	64.9 mn	16.8 n	22.6 b	1.3 hi	13.8 mn	16.1 h-j	10.0 n
Cheongja5 (Bl)	27.0 h	8.9 l	15.0 jk	26.4 mn	21.8 j-l	3.9 de	17.1 lm	12.8 jk	15.8 mn	14.2 ij	38.7 h-j	67.5 jk	18.0 g-j	19.9 n	1.9 d-i	14.1 lm	16.6 f-i	10.5 j-m
Cheongjakong (Bl)	24.2 o	8.8 m	14.4 n	25.8 pq	20.5 p	4.0 cd	17.1 j-l	13.1 g-i	15.7 n	14.4 hi	38.1 jk	67.0 kl	18.0 h-j	18.8 p	2.7 a-e	14.5 jk	17.8 b-e	10.3 lm
Cheongyeob1 (Bl)	23.4 p	8.5 n	14.6 mn	25.5 q	20.5 p	3.5 i-k	16.7 n	12.3 l	15.2 o	13.7 k	35.7 n	63.5 n	16.5 n	18.9 p	1.1 i	13.7 n	15.1 j	10.1 n
Daeheug (Bl)	26.6 h-j	9.2 gh	15.7 de	27.7 d-g	21.9 h-k	3.5 i-k	18.4 c	14.1 bc	16.8 c-f	15.7 c-e	40.3 de	72.6 b	19.1 bc	21.9 de	2.4 b-f	15.6 cd	17.1 c-h	11.1 d-f
Geomjeongkong1 (Bl)	28.4 e	9.6 bc	15.6 d-f	27.8 de	22.3 ef	3.8 ef	18.4 cd	14.0 b-d	17.0 b-e	15.9 c	41.3 c	72.1 bc	18.9 b-d	22.1 cd	2.4 b-g	15.7 bc	17.8 b-e	10.9 fg
Geomjeongkong2 (Bl)	26.6 ij	9.2 f-h	15.3 hi	27.2 h-j	22.0 f-i	3.8 fg	17.7 g	13.8 c-e	16.6 f-i	15.8 c	41.0 cd	70.8 c-f	18.8 b-e	21.2 h-j	2.3 c-h	15.3 ef	17.6 b-f	11.0 d-f
Geomjeongkong3 (Bl)	27.0 fg	9.5 de	15.4 f-h	27.4 f-h	22.2 e-g	3.6 hi	18.0 ef	13.6 ef	16.7 e-h	15.8 c	40.0 ef	70.3 d-g	18.4 e-g	21.4 f-h	1.8 e-i	15.5 c-e	17.3 c-g	10.9 fg
Geomjeongkong4 (Bl)	24.5 no	9.1 i-k	15.1 jk	26.8 kl	21.4 mn	3.5 jk	17.6 gh	13.3 fg	16.4 h-j	15.0 g	38.1 jk	66.3 k-m	18.1 g-i	19.7 n	2.8 a-d	14.7 ij	17.0 d-i	10.7 hi
Geomjeongkong5 (Bl)	27.3 f	9.5 cd	15.6 d-f	27.7 d-g	22.0 g-k	3.8 fg	18.1 de	13.7 de	16.8 c-g	15.8 c	41.4 c	71.2 b-f	18.7 c-f	22.2 c	2.4 b-g	15.6 c-e	18.0 a-d	11.1 de
Heugmi (Bl)	24.4 no	8.7 m	15.3 g-i	26.8 k-m	21.4 mn	3.6 h	17.2 i-l	13.2 gh	16.3 i-k	14.5 h	38.2 i-k	65.8 lm	17.5 kl	20.4 lm	1.6 f-i	14.6 i-k	17.2 c-g	10.5 jk
Heugsung (Bl)	26.7 g-i	9.2 hi	15.2 ij	26.9 j-l	21.4 mn	3.8 fg	17.7 fg	13.2 gh	16.6 f-i	15.0 g	39.2 f-h	70.2 e-g	18.1 g-i	21.0 ij	2.7 a-e	15.0 f-h	17.6 b-f	10.4 j-m
Ilpumgeomjeong2 (Bl)	25.5 l	9.1 h-k	15.9 c	27.9 de	22.0 g-j	4.0 cd	18.6 c	13.7 de	17.1 bc	15.7 cd	39.2 gh	68.7 g-j	18.3 f-h	20.7 kl	3.0 a-c	15.5 c-e	17.0 d-h	11.2 d
Ilpumgeomjeongkong (Bl)	26.4 jk	9.4 e-g	15.8 cd	27.6 e-h	22.1 e-h	4.0 cd	18.1 e	14.1 b	16.8 c-f	15.8 c	39.8 e-g	69.9 f-h	18.6 d-f	20.9 jk	3.0 a-c	15.7 bc	16.5 g-i	11.5 c
Jungmo3009 (Bl)	25.3 l	9.1 i-k	15.4 f-h	27.3 g-i	21.9 h-l	3.7 g	17.8 fg	14.0 b-d	16.6 f-i	15.4 ef	39.0 g-i	69.1 g-i	18.6 c-f	20.4 lm	2.4 b-g	15.5 c-e	16.6 f-i	11.0 ef

Jungmo3011 (Bl)	31.5 c	9.7 b	15.6 e-g	27.7 d-f	22.7 c	3.9 de	17.8 fg	13.8 c-e	16.5 g-i	15.8 c	41.8 bc	72.4 b	19.2 b	21.4 f-h	2.9 a-c	15.5 c-e	17.0 d-h	11.1 de
Seonheukkong (Bl)	26.8 g-i	9.4 ef	15.7 de	27.8 d-g	22.7 c	3.6 hi	18.1e	13.9 b-e	16.8 d-g	15.5 d-f	39.9 e-g	69.2 g-i	18.7 b-e	20.6 kl	2.7 a-e	15.3 d-f	16.9 e-i	11.2 de
Seoritae (Bl)	34.5 a	8.8 m	14.7 lm	25.8 pq	21.7 kl	3.8 fg	17.1 j-l	13.1 g-j	15.8 mn	14.5 hi	38.5 h-k	67.8 i-k	17.9 h-k	22.7 b	3.3 a-c	14.3 kl	17.4 c-g	10.4 k-m
Socheong (Bl)	23.2 p	8.8 lm	14.9 k	26.6 l-n	21.4 mn	4.1 b	17.4 h-j	13.3 fg	16.0 k-m	15.0 g	37.2 lm	65.0 mn	18.0 h-j	21.6 ef	2.6 a-f	14.6 ij	17.2 c-g	10.5 j-l
Socheong2 (Bl)	23.3 p	9.1 k	14.9 kl	26.4 m-o	21.9 i-l	3.6 h-j	17.0 lm	14.0 b-d	16.0 l-n	15.3 fg	37.6 kl	65.3 m	18.6 d-f	20.3 m	2.6 a-e	15.1 fg	16.0 ij	10.6 ij
Socheongja (Bl)	25.0 m	8.5 n	14.4 n	26.0 op	21.2 n	3.5 i-k	16.8 m	12.9 h-k	15.8 mn	14.4 h-j	37.2 lm	63.4 n	17.3 lm	19.3 o	2.9 a-d	14.1 l	16.5 f-i	10.3 m
Taecheong (Bl)	33.1 b	9.2 h-j	15.1 jk	26.4 no	22.0 g-k	3.6 hi	17.1 kl	13.3 fg	16.4 h-j	15.1 g	42.6 ab	71.9 b-d	18.8 b-e	23.6 a	3.6 a	14.8 g-i	18.1 a-c	10.5 jk
Tawonkong (Bl)	28.7 e	10.1 a	16.9 a	30.1 a	24.3 a	4.3 a	19.8 a	14.8 a	17.8 a	16.6 a	43.5 a	76.2 a	20.2 a	23.5 a	3.4 ab	16.6 a	18.4 ab	12.3 a
Wonheug (Bl)	30.7 d	10.0 a	16.4 b	29.6 b	23.6 b	4.1 b	19.3 b	14.7 a	17.6 a	16.3 b	43.0 a	75.6 a	20.2 a	22.6 b	2.8 a-d	16.7 a	18.9 a	12.3 a
Jinyul (Br)	27.4 f	9.6 bc	15.8 c-e	28.0 d	22.4 de	3.1 lm	18.6 c	14.0 b-d	17.2 b	15.9 c	40.2 de	71.7 b-e	18.7 b-e	20.9 jk	3.4 ab	16.0 b	16.8 e-i	11.5 c
Chungdu1 (Gr)	24.1 o	9.1 i-k	14.9 kl	26.6 l-n	21.3 n	3.2 l	17.4 hi	12.6 kl	15.7 n	14.3 h-j	36.4 mn	65.9 lm	16.9 mn	21.6 e-g	1.0 i	14.0 lm	16.9 e-i	10.4 k-m
Jungmo3005 (Gr)	26.1 k	9.7 b	15.8 c-e	28.5 c	22.6 cd	4.0 bc	18.4 c	14.1 bc	17.1 b-d	15.8 c	41.3 c	71.9 b-d	19.0 b-d	21.3 g-i	3.2 a-c	15.7 bc	16.7 f-h	11.8 b
LSD (5%)	0.35	0.12	0.23	0.39	0.27	0.08	0.26	0.34	0.32	0.29	0.87	1.59	0.44	0.32	1.02	0.34	1.06	0.16

Arg: arginine, His: histidine, Ile: isoleucine, Leu: leucine, Lys: lysine, Met: methionine, Phe: phenylalanine, Thre: threonine, Val: valine, Ala: alanine, Asp: asparagine, Glu: glutamine, Ser: serine, Amm: ammonium, Cys: cysteine, Gly: glycine, Pro: proline, Tyr: tyrosine. Bl, Br, and Gr, in the parentheses, after the name of genotypes indicate their seed coat color black, brown, and green, respectively. Mean values followed by different letters in the same column indicate significantly different at $p < 0.05$ (n= 2).

Supplementary Table S3. Fatty acid content (% of oil content) in the seeds of 29 soybean genotypes.

Genotype	Palmitic	Stearic	Oleic	Linoleic	Linolenic
Cheongja2 (Bl)	10.5 k-m	3.4 d-g	23.1 g-i	54.4 f-i	8.6 e
Cheongja3 (Bl)	10.1 no	3.2 g-j	20.5 k-m	58.2 ab	8.0 g
Cheongja4 (Bl)	10.9 f-j	3.5 de	25.7 de	52.7 jk	7.2 l
Cheongja5 (Bl)	11.8 c	3.4 d-h	16.5 no	58.6 ab	9.7 b
Cheongjakong (Bl)	10.2 no	3.2 h-k	25.5 d-f	54.0 g-j	7.2 l
Cheongyeob1 (Bl)	12.9 a	3.5 d	22.3 g-k	53.6 h-k	7.7 h
Daeheug (Bl)	12.4 b	3.2 g-j	28.4 c	50.2 l	5.8 q
Geomjeongkong1 (Bl)	10.7 j-l	3.3 d-i	29.7 c	49.6 lm	6.7 n
Geomjeongkong2 (Bl)	10.7 i-l	3.4 d-h	29.4 c	49.7 lm	6.9 m
Geomjeongkong3 (Bl)	10.7 h-j	3.1 i-l	21.9 i-k	55.6 d-g	8.7 e
Geomjeongkong4 (Bl)	11.0 e-h	3.5 d-f	23.4 f-i	54.7 e-h	7.5 jk
Geomjeongkong5 (Bl)	11.0 e-h	2.9 k-m	35.7 b	43.2 o	7.3 l
Heugmi (Bl)	11.0 d-g	4.1 bc	24.0 e-h	53.6 h-k	7.4 k
Heugsung (Bl)	11.9 c	3.1 i-k	29.0 c	48.1 m	8.0 g
Ilpumgeomjeong2 (Bl)	9.7 p	2.6 n	28.7 c	49.4 lm	9.6 b
Ilpumgeomjeongkong (Bl)	10.3 m-o	3.9 bc	34.3 b	46.0 n	5.6 r
Jungmo3009 (Bl)	10.8 g-j	3.3 e-i	19.7 lm	55.8 d-f	10.5 a
Jungmo3011 (Bl)	9.8 p	3.5 de	22.7 g-j	56.5 cd	7.6 ij
Seonheukkong (Bl)	10.1 o	2.8 l-n	38.8 a	42.2 o	6.1 p
Seoritae (Bl)	10.7 i-k	3.4 d-h	26.1 d	52.2 k	7.6 hi
Socheong (Bl)	11.0 d-f	2.8 mn	19.2 lm	57.5 a-c	9.5 c
Socheong2 (Bl)	10.4 mn	3.4 d-h	18.5 mn	58.7a	9.1 d
Socheongja (Bl)	11.7 c	3.8 c	21.9 h-k	54.1 g-j	8.5 f
Taecheong (Bl)	11.2 d	3.2 f-i	20.8 j-l	57.6 a-c	7.2 l
Tawonkong (Bl)	11.2 de	4.1 ab	19.7 lm	57.0 b-d	8.0 g
Wonheug (Bl)	12.0 c	4.3 a	16.1 o	57.2 a-d	10.5 a
Jinyul (Br)	10.4 lm	3.0 j-m	34.7 b	45.7 n	6.3 o
Chungdu1 (Gr)	10.9 f-i	3.4 d-g	24.0 e-g	52.9 i-k	8.7 e
Jungmo3005 (Gr)	10.5 k-m	3.1 i-l	22.6 g-k	56.2 c-e	7.7 h
LSD (5%)	0.25	0.25	2.09	1.65	0.12

Bl, Br, and Gr, in the parentheses, after the name of genotypes indicate their seed coat color black, brown, and green, respectively. Mean values followed by different letters in the same column indicate significantly different at $p < 0.05$ (n= 2).

Supplementary Table S4. Free sugar concentration (mg/g) in the seeds of 29 soybean genotypes.

Genotype	Stachyose	Raffinose	Sucrose	Glucose	Galactose	Fructose
Cheongja2 (Bl)	21.5 gh	7.5 i	49.3 de	4.2 g	1.9 e-i	1.7 bc
Cheongja3 (Bl)	18.7 mn	8.0 de	43.9 i	4.5 f	2.1 b-e	1.5 c-e
Cheongja4 (Bl)	19.1 lm	7.9 fg	51.0 c	5.4 b	2.1 b-e	1.7 bc
Cheongja5 (Bl)	20.5 jk	6.7 lm	45.2 g	4.2 gh	2.2 bc	1.6 cd
Cheongjakong (Bl)	23.7 d	9.1 b	50.9 c	4.0 i	2.4 a	1.4 de
Cheongyeob1 (Bl)	22.0 fg	6.9 kl	45.0 gh	4.6 f	1.5 jk	1.3 d-f
Daeheug (Bl)	20.3 k	6.0 o	36.9 o	6.0 a	1.4 k	2.7 a
Geomjeongkong1 (Bl)	26.5 b	6.4 n	53.1 b	3.3 l	1.8 hi	1.2 ef
Geomjeongkong2 (Bl)	20.3 k	7.9 ef	46.9 f	4.8 e	1.9 f-i	0.8 gh
Geomjeongkong3 (Bl)	21.2 hi	7.8 f-h	49.6 d	4.9 de	2.2 bc	1.3 d-f
Geomjeongkong4 (Bl)	21.0 ij	8.2 cd	47.1 f	4.0 i	2.1 b-d	1.0 fg
Geomjeongkong5 (Bl)	17.9 o	6.9 k	43.7 ij	5.1 cd	1.7 ij	0.9 gh
Heugmi (Bl)	24.4 c	5.3 p	38.5 n	3.0 n	2.1 b-e	1.3 d-f
Heugsung (Bl)	21.2 hi	7.9 ef	48.5 e	3.9 ij	2.1 b-f	2.0 b
Ilpumgeomjeong2 (Bl)	28.2 a	7.7 gh	54.6 a	4.0 hi	2.1 b-e	1.9 b
Ilpumgeomjeongkong (Bl)	22.2 f	6.3 n	40.1 m	3.4 kl	1.8 g-i	ND
Jungmo3009 (Bl)	22.7 e	7.5 i	52.5 b	4.6 f	1.7 i	0.8 gh
Jungmo3011 (Bl)	19.4 l	9.3 a	44.1 hi	4.3 g	2.0 d-h	ND
Seonheukkong (Bl)	27.7 a	7.0 k	35.9 p	2.9 n	2.0 d-h	ND
Seoritae (Bl)	22.1 f	7.2 j	45.2 g	4.2 g	2.0 d-h	1.2 ef
Socheong (Bl)	24.1 cd	6.7 m	42.7 jk	3.8 j	2.0 d-h	0.8 gh
Socheong2 (Bl)	26.7 b	7.7 h	30.7 r	3.1 m	2.0 c-g	ND
Socheongja (Bl)	21.1 hi	7.7 h	38.3 n	5.0 de	2.2 b	1.2 ef
Taecheong (Bl)	18.3 no	9.3 a	47.3 f	5.2 c	2.5 a	1.5 c-e
Tawonkong (Bl)	22.1 f	6.6 m	28.8 s	3.5 k	2.1 b-f	0.7h
Wonheug (Bl)	22.3 ef	8.3 c	31.7 q	4.0 hi	1.7 ij	ND
Jinyul (Br)	26.9 b	8.2 c	41.8 kl	2.9 n	2.2 b-d	0.8 gh
Chungdu1 (Gr)	24.1 cd	7.56 i	41.0 lm	3.9 ij	1.8 g-i	0.9 gh
Jungmo3005 (Gr)	21.0 ij	7.3 ij	40.7 m	3.3 l	1.9 f-i	ND
LSD (5%)	0.47	0.17	0.96	0.15	0.21	0.27

Bl, Br, and Gr, in the parentheses, after the name of genotypes indicate their seed coat color black, brown, and green, respectively. Mean values followed by different letters in the same column indicate significantly different at $p < 0.05$ (n= 3). ND: non-detectable.

Supplementary Table S5. Isoflavones content (μg/g) in the seeds of 29 soybean genotypes.

Genotype	Di	Gly	Gi	Mdi	Mgly	Mgi	AcDi	AcGly	De	Gle	AcGi	Ge
Cheongja2 (Bl)	120.3 c-e	48.2 b-d	211.6 a-c	693.6 d-g	92.0 gh	1006.4 c-f	24.3 b-d	ND	111.6 b-g	17.9 d-h	6.4 c-f	96.5 a-c
Cheongja3 (Bl)	70.1 g-i	15.8 kl	112.4 i-k	595.9 f-h	50.7 mn	900.0 f-h	ND	7.4 c-e	50.8 g-k	10.0 gh	ND	55.5 f-k
Cheongja4 (Bl)	81.6 f-h	32.4 ef	132.4 g-j	619.9 f-h	71.6 i-l	810.3 h-j	ND	ND	44.4 h-k	23.5 c-g	ND	50.3 g-k
Cheongja5 (Bl)	192.2 a	34.9 e	207.6 a-d	1282.5 a	59.2 k-m	1138.3 a-c	ND	ND	138.3 bc	12.3 f-h	ND	74.9 b-i
Cheongjakong (Bl)	96.9 c-g	19.7 i-k	143.6 g-j	593.8 f-h	61.4 j-m	866.8 f-i	22.4 de	5.9 e-h	82.3 c-i	11.4 f-h	5.7 d-g	72.5 b-i
Cheongyeob1 (Bl)	73.4 g-i	20.4 h-k	157.8 e-g	393.7 j	58.1 k-m	779.0 h-j	23.6 b-d	6.4 d-g	41.8 h-k	12.4 f-h	5.5 d-h	58.5 e-k
Daeheug (Bl)	85.9 fg	31.1 ef	146.6 g-j	514.0 h-j	97.6 fg	714.4 i-k	22.8 c-e	3.5 g-j	62.4 e-i	15.5 f-h	6.2 d-f	59.8 d-k
Geomjeongkong1 (Bl)	46.0 i-k	17.6 j-l	50.5 l	423.9 ij	77.6 h-j	446.9 n	23.8 b-d	3.3 h-j	29.1 h-k	13.4 f-h	12.7 b	34.7 j-l
Geomjeongkong2 (Bl)	99.2 c-g	23.2 g-j	156.6 e-h	734.7 d-f	83.2 g-i	1163.2 ab	22.8 c-e	8.8 cd	75.8 d-i	16.7 e-h	6.9 c-f	78.3 b-g
Geomjeongkong3 (Bl)	88.4 e-g	42.6 d	107.9 jk	617.9 f-h	134.3 bc	774.6 h-j	20.6 e	ND	87.5 c-h	39.6 b	3.7 f-i	65.6 d-j
Geomjeongkong4 (Bl)	156.9 b	29.8 e-g	165.0 d-g	1058.4 b	115.9 de	1059.3 b-d	23.3 b-d	2.7 ij	154.3 b	23.6 c-g	4.2 e-i	83.2 b-f
Geomjeongkong5 (Bl)	82.0 f-h	12.2 l	128.5 g-j	500.2 h-j	49.6 mn	744.5 i-k	24.4 b-d	13.5 b	85.9 c-h	18.0 d-h	9.8 bc	72.6 b-i
Heugmi (Bl)	96.6 c-g	44.5 cd	113.8 h-k	554.4 g-i	72.8 i-k	605.0 k-m	22.9 c-e	ND	87.1 c-h	23.8 c-g	2.4 g-i	58.6 e-k
Heugsung (Bl)	98.9 c-g	20.4 h-k	113.1 j-i	594.2 f-h	65.8 j-m	661.3 j-l	22.5 de	1.9 j	55.5 f-j	15.2 f-h	3.7 f-i	50.1 g-k
Ilpumgeomjeong2 (Bl)	97.5 c-g	26.5 f-i	195.9 b-e	673.0 e-g	76.2 h-j	1252.0 a	24.0 b-d	ND	112.3 b-f	36.1 bc	8.5 cd	121.2 a
Ilpumgeomjeongkong (Bl)	122.1 cd	34.6 e	133.6 g-j	917.9 bc	130.7 b-d	901.7 e-h	25.2 a-c	4.6 e-j	137.0 bc	32.6 b-d	8.3 cd	78.1 b-h
Jungmo3009 (Bl)	156.2 b	31.7 ef	247.5 a	914.3 bc	75.7 h-j	1246.2 a	25.6 ab	5.7 e-i	125.9 b-d	18.7 d-h	8.4 cd	99.6 ab
Jungmo3011 (Bl)	49.7 h-k	16.5 j-l	77.0 kl	371.2 jk	31.6 o	454.7 mn	ND	4.2 f-j	1.3 jk	ND	ND	19.5 l
Seonheukkong (Bl)	27.7 k	16.9 j-l	75.9 kl	211.7 l	77.0 h-j	517.8 l-n	ND	23.1 a	0.3 jk	15.2 f-h	2.2 hi	44.9 i-l
Seoritae (Bl)	110.1 c-f	47.9 b-d	160.1 e-g	731.8 d-f	112.3 ef	822.0 g-i	ND	ND	42.3 h-k	21.9 c-h	ND	36.7 j-l
Socheong (Bl)	197.8 a	63.8 a	151.5 f-i	1404.5 a	132.5 bc	1055.6 b-e	27.2 a	ND	253.6 a	31.4 b-e	6.2 d-f	90.5 b-d
Socheong2 (Bl)	94.0 d-g	54.8 b	168.7 c-g	828.4 cd	212.4 a	1203.8 ab	ND	4.8 e-j	52.2 f-k	63.1 a	ND	72.8 b-i
Socheongja (Bl)	96.5 c-g	43.0 d	132.8 g-j	726.2 d-f	120.3 c-e	855.3 f-i	ND	9.4 c	55.8 f-j	23.3 c-g	ND	54.2 f-k
Taecheong (Bl)	34.6 k	21.3 h-k	51.5 l	248.8 kl	41.2 no	287.3 o	ND	7.0 c-f	ND	7.6 h	ND	18.9 l
Tawonkong (Bl)	36.4 jk	32.8 ef	78.8 kl	249.2 kl	54.7 mn	469.0 mn	ND	ND	ND	18.9 d-h	6.8 c-f	31.2 kl
Wonheug (Bl)	68.1 g-j	47.5 cd	136.6 g-j	438.3 ij	121.8 c-e	858.4 f-i	ND	ND	22.5 i-k	64.7 a	20.4 a	57.7 e-k
Jinyul (Br)	32.2 k	44.0 cd	70.8 kl	226.4 l	108.3 ef	438.3 no	ND	ND	ND	24.1 c-g	1.5 i	46.2 h-l
Chungdu1 (Gr)	158.0 b	50.9 bc	216.4 ab	808.7 c-e	144.6 b	1008.9 c-f	23.7 b-d	5.3 e-i	117.5 b-e	32.6 b-d	6.8 c-f	74.2 b-i
Jungmo3005 (Gr)	127.6 bc	27.4 f-h	192.9 b-f	680.2 e-g	55.3 l-n	967.6 d-g	25.5 ab	ND	122.7 b-e	26.6 b-f	7.2 c-e	87.8 b-e
LSD (5%)	33.41	7.14	43.45	144.47	16.30	154.01	2.50	2.58	51.17	12.83	3.46	26.10

Di: daidzin, Gly: glycitin, Gi: genistin, Mdi: malonyldaidzin, Mgly: malonylglycitin, Mgi: malonylgenistin, AcDi: acetyldaidzin, AcGly: acetylglycitin, De: daidzein, Gle: glycitein, AcGi: acetylgenistin, and Ge: genistein. Bl, Br, and Gr, in the parentheses, after the name of genotypes indicate their seed coat color black, brown, and green, respectively. ND: non-detectable. Mean values followed by different letters in the same column indicate significantly different at $p < 0.05$ (n= 3).

Supplementary Table S6. Anthocyanin content (µg/g seed coat) in the seeds of 29 soybean genotypes.

Genotype	Dp3glc	Cy3gal	Cy3glc	Pt3glc	Pg3glc	Pn3glc
Cheongja2 (Bl)	3015.8 a	180.7 d-g	12957.0 c-g	1244.6 a	216.5 cd	338.8 f-i
Cheongja3 (Bl)	1540.0 e-g	173.8 e-g	9044.0 i-k	733.9 b	335.7 c	337.1 f-i
Cheongja4 (Bl)	1185.1 fg	178.8 e-g	10080.0 h-j	640.4 bc	207.2 cd	331.7 g-i
Cheongja5 (Bl)	ND	258.2 ab	16483.0 ab	182.2 d	213.5 cd	647.5 a
Cheongjakong (Bl)	1998.6 c-e	163.1 fg	10470.0 g-j	551.1 bc	163.2 d	301.7 hi
Cheongyeob1 (Bl)	ND	185.6 c-f	13031.0 c-g	179.2 d	131.9 d	368.9 d-g
Daeheug (Bl)	2616.5 ab	191.9 c-f	12867.0 c-h	684.4 b	152.6 d	317.3 g-i
Geomjeongkong1 (Bl)	ND	114.2 h	3318.0 n	ND	144.2 d	307.3 hi
Geomjeongkong2 (Bl)	2641.4 ab	273.1 a	17414.0 a	1001.3 a	161.8 d	364.3 d-g
Geomjeongkong3 (Bl)	3145.1 a	189.6 c-f	13404.0 c-f	566.1 bc	132.0 d	295.5 hi
Geomjeongkong4 (Bl)	1691.3 d-g	181.7 d-f	10619.0 f-j	571.0 bc	116.9 d	292.5 i
Geomjeongkong5 (Bl)	2436.9 bc	145.3 f-h	5396.0 l-n	999.6 a	527.3 b	289.6 i
Heugmi (Bl)	1784.7 d-f	155.4 f-h	8199.0 j-l	612.5 bc	146.3 d	297.7 hi
Heugsung (Bl)	1661.9 d-g	132.8 gh	6210.0 k-m	1006.0 a	805.9 a	326.9 g-i
Ilpumgeomjeong2 (Bl)	2767.1 ab	166.2 e-g	11527.0 e-i	707.5 b	130.9 d	294.0 hi
Ilpumgeomjeongkong (Bl)	2214.6 b-d	152.9 f-h	10527.0 g-j	595.1 bc	141.6 d	306.4 hi
Jungmo3009 (Bl)	ND	213.7 b-e	15404.0 a-c	186.0 d	472.6 b	501.6 b
Jungmo3011 (Bl)	1121.8 g	187.2 c-f	12523.0 d-h	425.4 cd	162.9 d	317.6 g-i
Seonheukkong (Bl)	ND	173.0 e-g	10464.0 g-j	184.5 d	158.5 d	390.4 c-e
Seoritae (Bl)	1209.5 fg	168.5 e-g	4542.0 mn	423.3 cd	ND	317.6 g-i
Socheong (Bl)	ND	231.2 a-c	13898.0 b-e	ND	ND	472.4 b
Socheong2 (Bl)	ND	242.6 ab	15257.0 a-d	ND	165.5 d	413.5 cd
Socheongja (Bl)	170.2 h	227.1 b-d	14305.0 b-e	182.0 d	131.9 d	420.8 c
Taecheong (Bl)	1573.8 e-g	193.2 c-f	13141.0 c-g	407.5 cd	133.9 d	346.9 e-h
Tawonkong (Bl)	1427.5 e-g	132.5 gh	3063.0 n	1136.0 a	164.3 d	384.5 c-f
Wonheug (Bl)	1594.8 e-g	151.5 f-h	6436.0 k-m	707.4 b	ND	333.8 f-i
Jinyul (Br)	ND	ND	ND	ND	ND	ND
Chungdu1 (Gr)	ND	ND	ND	ND	ND	ND
Jungmo3005 (Gr)	ND	ND	ND	ND	ND	ND
LSD (5%)	517.50	40.07	2845.50	222.60	136.50	44.63

Dp3glc: delphinidin-3-glucoside, Cy3gal: cyanidin-3-galactoside, Cy3glc: cyanidin-3-glucoside, Pt3glc: petunidin-3-glucoside, Pg3glc: pelargonidin-3-glucoside, Pn3glc: peonidin-3-glucoside. Bl, Br, and Gr, in the parentheses, after the name of genotypes indicate their seed coat color black, brown, and green, respectively. ND: non-detectable. Mean values followed by different letters in the same column indicate significantly different at $p < 0.05$ (n= 3).
